# ALYREF mediates RNA m^5^C modification to promote hepatocellular carcinoma progression

**DOI:** 10.1038/s41392-023-01395-7

**Published:** 2023-03-18

**Authors:** Chen Xue, Xinyu Gu, Qiuxian Zheng, Qingmiao Shi, Xin Yuan, Yuanshuai Su, Junjun Jia, Jianwen Jiang, Juan Lu, Lanjuan Li

**Affiliations:** 1 State Key Laboratory for Diagnosis and Treatment of Infectious Diseases, National Clinical Research Center for Infectious Diseases, National Medical Center for Infectious Diseases, Collaborative Innovation Center for Diagnosis and Treatment of Infectious Diseases, The First Affiliated Hospital, Zhejiang University School of Medicine, Hangzhou, Zhejiang China; 2 Division of Hepatobiliary and Pancreatic Surgery, Department of Surgery, The First Affiliated Hospital, Zhejiang University School of Medicine, Hangzhou, Zhejiang China

**Keywords:** Epigenomics, Gastrointestinal cancer

**Dear Editor,**

The incidence of hepatocellular carcinoma (HCC) is increasing in many countries due to hepatic virus infection and unhealthy diet habits, and the unsatisfactory overall survival rate calls for novel treatments with higher efficacy.^1^ Recent studies have shown that RNA modifications are likely to participate in the progression of HCC,^2^ suggesting new methods for tumor growth control. Specifically, 5-methylcytosine (m^5^C) is one of the most interesting RNA modification types, as accumulating evidence suggests its role in the promotion of HCC tumorigenesis.^3^ The RNA methyltransferase Aly/REF export factor (ALYREF) is considered one type of “reader” protein located in the nucleus that recognizes and binds directly with m^5^C sites in RNA and facilitates the export of RNA from the nucleus to the cytoplasm.^4^ Notably, ALYREF is considered a promising target for diagnosis and prognosis prediction.^5^ However, until now, the low number of related studies has limited the understanding of the mechanism of the HCC-promoting effects of ALYREF. To further elucidate the oncogenic roles of ALYREF in HCC, we assessed the expression levels of ALYREF in clinical samples and HCC cell lines and explored the effects of ALYREF deficiency by both in vitro and in vivo experiments and m^5^C-methylated RNA immunoprecipitation sequencing (m^5^C-MeRIP-Seq).

First, we collected HCC tissues and adjacent non-tumor tissues from HCC patients and detected the expression levels of thirteen m^5^C regulators, including NSUN1–7, DNMT1–2, DNMT3A, DNMT3B, ALYREF, and TET2, using RNA sequencing (RNA-seq) (Fig. 1a). The ALYREF expression level was also assessed using Western blotting (Fig. 1b) and quantitative reverse transcriptase PCR (qRT-PCR) (Fig. 1c). As expected, the expression level of ALYREF was significantly higher in HCC tissues than in adjacent non-tumor tissues, which is consistent with the results of previous studies.^4^ We also chose four different HCC cell lines and compared the expression levels of ALYREF with those in the normal liver cell line LO2 (Supplementary Fig. 1a), and elevated ALYREF expression was observed in all four tested HCC cell lines.

**Fig. 1:**
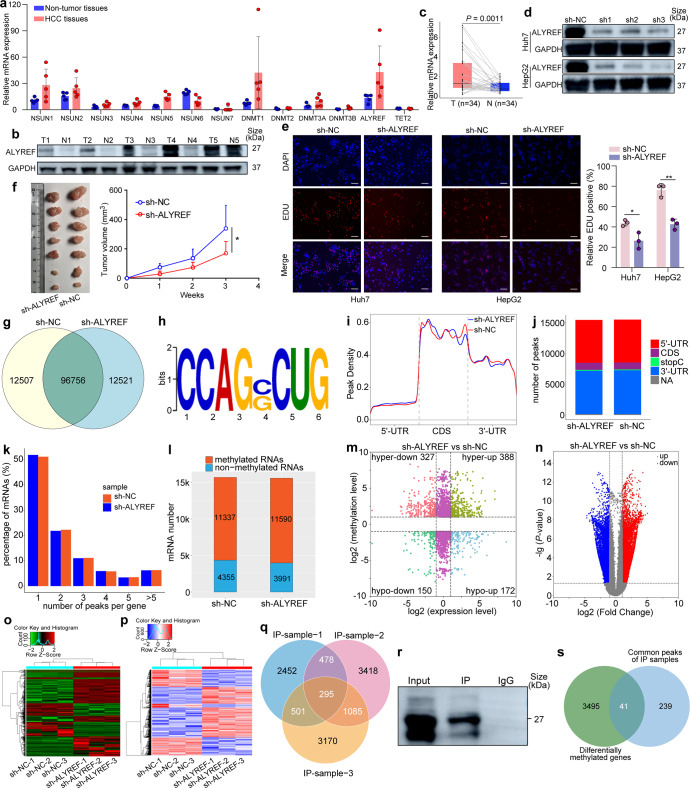
ALYREF mediates RNA m^**5**^C modification to promote hepatocellular carcinoma progression. **a** The expression levels of m^5^C-related genes, including NSUN1–7, DNMT1–2, DNMT3a, DNMT3b, ALYREF, and TET2, were assessed by RNA-seq in 5 pairs of HCC tissues and normal liver tissues. **b** Western blotting showed elevated ALYREF expression in tumors in 5 pairs of HCC tissues and adjacent tissues from HCC patients. **c** qRT-PCR demonstrated increased ALYREF levels in HCC tissues compared with adjacent tissues (*n* = 34, *P* = 0.0011). **d** Western blotting proved the efficiency of ALYREF knockdown in Huh7 and HepG2 cells by transfection with sh-NC and sh-ALYREF lentivirus. **e** EdU assay showed the impaired vitality of ALYREF-deficient cells. Scale bar, 100 μm. **f** In vivo experiments showed that ALYREF-deficient tumors had a smaller size and a lighter weight. **g** m^5^C-MeRIP-Seq illustrated 109,263 and 109,277 m^5^C sites of mRNA in sh-NC and sh-ALYREF Huh7 cells, respectively. Additionally, the two groups shared most methylated mRNA sites. **h** The most conserved motif was CCAGRCUG (R = C/G) in both sh-NC and sh-ALYREF Huh7 cells. **i**, **j** In both sh-NC and sh-ALYREF Huh7 cells, the CDS region had the highest peak density, followed by the 3’-UTR and 5’-UTR, but the number of peaks in the CDS region was less than that in the 3’-UTR and 5’-UTR regions. **k** Most mRNAs possessed only one methylation peak in both sh-NC and sh-ALYREF Huh7 cells. **l** A total of 11,337 and 11,590 methylated genes and 4355 and 3991 nonmethylated genes were found in sh-NC and sh-ALYREF Huh7 cells, respectively. **m** A four-quadrant diagram displaying alterations in gene methylation and expression, in sh-ALYREF Huh7 cells. **n** A volcano chart displaying changes in gene expression levels in sh-ALYREF cells compared to sh-NC cells. **o** A heatmap displaying the results of transcriptome hierarchical clustering analysis in sh-NC and sh-ALYREF Huh7 cells. **p** A heatmap showing the results of hierarchical clustering analysis of differentially methylated mRNA in sh-NC and sh-ALYREF Huh7 cells. **q**, **r** RIP analysis revealed 295 ALYREF-binding genes. Western blotting was used to confirm the RIP results. **s** The Venn diagram of differentially methylated genes after ALYREF knockdown and the genes that were able to directly bind to ALYREF protein. Data are presented as mean ± SEMs. A value of *P* < 0.05 was considered as statistically significant. *P < 0.05

Next, the effect of ALYREF deficiency on Huh7 and HepG2 cells was investigated. Three types of short hairpin RNA (shRNA) were used for ALYREF knockdown, and knockdown efficiency was validated by Western blotting (Fig. 1d), qRT-PCR (Supplementary Fig. 1b) and cell-based fluorescence assays (Supplementary Fig. 1c). We then performed a 5-ethynyl-20-deoxyuridine (EdU) assay (Fig. 1e), flow cytometry analysis (Supplementary Fig. 2a) and a colony formation assay (Supplementary Fig. 2b) on both sh-NC and sh-ALYREF cells. The results showed that ALYREF knockdown could significantly inhibit Huh7 and HepG2 cell growth while increase the apoptosis rate of HCC cells. Similar results were observed in SK-Hep-1 and Hep-3B cell lines (Supplementary Fig. 3). Additionally, BALB/c nude mice that received sh-ALYREF Huh7 cell injection developed smaller and lighter tumors than the sh-NC group after three weeks of tumor cell injection (Fig. 1f and Supplementary Fig. 2c), indicating the tumor-suppressing effect of ALYREF deficiency in vivo. Immunohistochemical (IHC) staining of HCC tissue sections using antibodies against ALYREF, Ki67, and PCNA revealed that sh-ALYREF significantly reduced the expression of ALYREF and the proliferation activity of HCC cells (Supplementary Fig. 2d).

To further explore the potential molecular mechanism of ALYREF in RNA m^5^C modification, we then performed m^5^C-MeRIP-Seq on both sh-NC and sh-ALYREF Huh7 and HepG2 cells. A total of 109,263 and 109,277 m^5^C sites of mRNA were found in sh-NC and sh-ALYREF Huh7 cells, respectively, and the two groups shared a majority of m^5^C-methylated mRNA sites (Fig. 1g). The most conserved motif, namely, CCAGRCUG (R = C/G), was the same in both groups, indicating that ALYREF deficiency did not cause alterations to the most frequently m^5^C-methylated motifs (Fig. 1h). In Huh7 cells in both the sh-NC and sh-ALYREF groups, the coding DNA sequence (CDS) region had the highest peak density, followed by the 3’-untranslated regions (3’-UTR) and 5’-UTR (Fig. 1i), but the number of peaks in the CDS region was less than that in the 3’-UTR and 5’-UTR regions (Fig. 1j). Moreover, the majority of mRNA possessed only one methylation peak in both groups (Fig. 1k), and a total of 11,337 and 11,590 methylated mRNA and 4355 and 3991 nonmethylated mRNA were found in sh-NC and sh-ALYREF Huh7 cells, respectively (Fig. 1l).

To determine whether ALYREF-associated m^5^C methylation affects mRNA expression, a four-quadrant diagram (Fig. 1m) and a volcano plot (Fig. 1n) were used to represent differently expressed mRNAs and m^5^C methylation sites. Two heatmaps showed the results of hierarchical clustering of differently expressed mRNAs by transcriptome analysis (Fig. 1o) and differentially methylated mRNAs (Fig. 1p) in sh-NC and sh-ALYREF cells. Gene set enrichment analysis (GSEA) was performed on genes associated with ALYREF and suggested that ALYREF plays a role in JAK-STAT signaling pathway, MAPK signaling pathway, TGF-β signaling pathway, Toll-like receptor signaling pathway, ubiquitin-mediated proteolysis, and the WNT signaling pathway (Supplementary Fig. 4). Similar analysis was also performed for circRNAs and lncRNAs in both Huh7-sh-NC and Huh7-sh-ALYREF cells (Supplementary Fig. 5). The m^5^C-MeRIP-Seq results of mRNAs, lncRNAs, and circRNAs in sh-NC and sh-ALYREF HepG2 cells were also analyzed (Supplementary Fig. 6 and Supplementary Fig. 7).

In light of our findings, we conducted RNA immunoprecipitation (RIP) to identify target genes that might bind to ALYREF in Huh7 cells. The results showed that a total of 295 peaks were targeted by ALYREF from IP samples based on three independent replicates (Fig. 1q). The protein products were eluted and assessed by Western blotting (Fig. 1r). Kyoto Encyclopedia of Genes and Genomes (KEGG) and Gene Ontology (GO) enrichment analyses were also utilized to determine the biofunctions of these ALYREF-binding genes. The results showed that ALYREF-binding genes were significantly enriched in the cell cycle, necroptosis, PD-L1 expression and PD-1 checkpoint pathway, hepatocellular carcinoma, autophagy, adherens junction, etc. (Supplementary Fig. 8). Then, we compared the identified genes of m^5^C-MeRIP-Seq and RIP and discovered that 41 genes were both differentially methylated after ALYREF knockdown and was able to directly bind to the ALYREF protein (Fig. 1s). Taken together, these results suggested that ALYREF takes part in HCC progression through directly binding to target genes by m^5^C modification.

In summary, our results increase the understanding of the role that ALYREF plays in HCC from a novel and broader perspective. The abundance of ALYREF in tumor tissues and HCC cell lines, along with the tumor-suppressive effects of ALYREF deficiency in vitro and in vivo, confirmed the oncogenic roles of ALYREF in HCC development. Mechanistically, our study suggests that ALYREF promotes HCC by regulating methylation levels of target genes. However, the axis of ALYREF-m^5^C-targets needs to be fully characterized in the further studies. Hopefully, our study will provide novel insight into ALYREF-based targeted therapy for HCC.

## Supplementary information

SUPPLEMENTAL MATERIAL

## Data Availability

All datasets associated with this study are available from the corresponding author upon reasonable request. The RNA-seq data can be accessed in BioProject database (ID: PRJNA862293). Transcriptome data and RNA methylation data are available in GEO DataSets GSE221793.
