# Supplementary material for: ALYREF mediates RNA m5C modification to promote hepatocellular carcinoma progression
Source: Signal Transduct Target Ther. 2023 Mar 18;8:130. doi: 10.1038/s41392-023-01395-7 (PMC10024699; doi:10.1038/s41392-023-01395-7)
Supplement: Supplementary file 1 — SUPPLEMENTAL MATERIAL [file 41392_2023_1395_MOESM1_ESM.docx]

**Supplemental Materials and Figures for**

**ALYREF Mediates RNA m^5^C Modification to Promote Hepatocellular Carcinoma Progression**

Chen Xue^1#^, Xinyu Gu^1#^, Qiuxian Zheng^1#^, Qingmiao Shi^1#^, Xin Yuan^1^, Yuanshuai Su^1^, Junjun Jia^2^, Jianwen Jiang^1^, Juan Lu^1*^, Lanjuan Li^1*^

^1^ State Key Laboratory for Diagnosis and Treatment of Infectious Diseases, National Clinical Research Center for Infectious Diseases, National Medical Center for Infectious Diseases, Collaborative Innovation Center for Diagnosis and Treatment of Infectious Diseases, The First Affiliated Hospital, Zhejiang University School of Medicine, Hangzhou, Zhejiang, China.

^2^ Division of Hepatobiliary and Pancreatic Surgery, Department of Surgery, The First Affiliated Hospital, Zhejiang University School of Medicine, Hangzhou, Zhejiang, China.

^#^These authors contributed equally: Chen Xue, Xinyu Gu, Qiuxian Zheng, and Qingmiao Shi

***Corresponding author**

**Lanjuan Li & Juan Lu,**

State Key Laboratory for Diagnosis and Treatment of Infectious Diseases, The First Affiliated Hospital, Zhejiang University School of Medicine, No. 79 Qingchun Road, Shangcheng District, Hangzhou, Zhejiang 310003, China. Tel: 86 0571-87236459; Email: ljli@zju.edu.cn (Lanjuan Li), lujuanzju@zju.edu.cn (Juan Lu).

**This file includes:**

Materials and Methods

Supplementary Figures 1 to 8

References

**Materials and methods**

***Patient data***

A total of 34 paired human HCC tissues and adjacent tissues were obtained from patients of The First Affiliated Hospital, Zhejiang University School of Medicine. All patients provided written informed consent**.** The use of human samples was approved by the ethics committee of the First Affiliated Hospital of Zhejiang University School of Medicine (IIT20210168B-R1) ^1^. All specimens were immediately snap-frozen in liquid nitrogen after surgical removal.

***Histological and Immunohistochemistry***

Fresh liver tissues were fixed in paraformaldehyde and embedded with paraffin. Hematoxylin and eosin (H&E) and immunohistochemical staining of liver sections was performed according to standard procedure. Paraffin-embedded liver sections were deparaffinized, hydrated, and incubated with primary antibody against ALYREF (Abcam, Cambridge, UK), Ki67 (Abcam, Cambridge, UK), and PCNA (Abcam, Cambridge, UK), respectively. Next, liver tissue sections were incubated with secondary antibody. The positive cells of immunohistochemical staining were evaluated using ImageJ IHC profiler (https://imagej.nih.gov/ij/). Liver sections were independently scored by two different pathologists who were blind to this study.

***Cell culture, transfection and generation of ALYREF-deficient HCC cells***

The human HCC cell lines HepG2 and Huh7 were obtained from FuHeng Biology (Shanghai, China). Cells were cultured in Dulbecco’s modified Eagle medium (DMEM) with 10% fetal bovine serum (FBS, Gimini, Calabasas, CA, USA). All cell lines were maintained at 37 °C with a humidified 5% CO2 atmosphere. Short hairpin RNAs (shRNAs) targeting ALYREF were purchased from Genomeditech Biotechnology (Shanghai, China). The shRNAs targeting ALYREF and the negative control were synthesized and cloned into the pGLVH1/GFP/Puro vector. We used 4 μg/mL puromycin (MedChemExpress, Cat. HY-K1057) for one week to obtain stably transfected HCC cell lines.

***qRT-PCR***

Total RNA was isolated and purified from target tissues and cell lines using TRIzol reagent (Invitrogen, USA) according to the manufacturer’s instructions. Complementary DNA (cDNA) was synthesized from 1 μg of total RNA by reverse transcription using PrimeScript reverse transcriptase (Takara). qRT-PCR was performed using a standard SYBR Green PCR Kit (Toyobo, Osaka, Japan) in the StepOnePlus Real-Time PCR System (ThermoFisher) ^2^. The forward (F) and reverse (R) primer sequences used for the amplification of ALYREF-R were TCTGGTCGCAGCTTAGGAAC and ALYREF-F were TGCCACCTCTGTTTACGCTC, GAPDH-R were GTCATGAGTCCTTCCACGATACC and GAPDH-F were GGAGTCCACTGGCGTCTTCA. The 2^–ΔΔCt^ method was used to calculate fold changes in expression normalized to the level of GAPDH.

***Western blotting***

A cell lysis reagent (Beyotime Biotechnology, Shanghai, China) containing a protease inhibitor and phosphatase inhibitor (Servicebio, Wuhan, China) was added to each group of cells. The concentration of extracted proteins was quantified using a BCA protein quantification kit (Beyotime, Shanghai, China). Equivalent amounts of proteins were resolved by sodium dodecyl sulfate‒polyacrylamide gel electrophoresis and transferred onto polyvinylidene fluoride membranes. The membranes were blocked using 5% skimmed milk in Tris-buffered saline containing 0.1% Tween 20 (TBST) for 2 h and incubated with primary antibodies (rabbit anti-Aly/Ref antibody (Abcam, ab202894)) in a shaking incubator at 4°C overnight. GAPDH (CST, 5174) antibodies were used as controls. After being washed with Tris-buffered saline with Tween-20 (TBST) 3 times, the proteins were incubated with secondary antibodies (Servicebio, Wuhan, China). The immunoblots were analyzed using a Bio-Rad imaging system.

***5-Ethynyl-20-deoxyuridine (EdU) assay and colony formation assay***

EdU staining was performed according to the manufacturers’ instructions. The cells were fixed in 4% methanol and cultured with 0.5% Triton X-100 for 10 minutes, and 400 μL of 1× ApollorR was then added. DAPI was used for cell staining for 30 minutes, after which the number of EdU-positive cells was calculated. In the colony formation assay, a total of 1000 cells/well were seeded in 6-well culture plates and cultivated for 14 days. The cell colonies were fixed with 0.1% crystal violet and counted by ImageJ software.

***Mouse strains and maintenance***

Four- to six-week-old male BALB/c nude mice were purchased from Shanghai Laboratory Animal Co. (SLAC), Ltd., and sh-NC and sh-ALYREF cells were injected into the backs of eight-week-old mice. The volume of tumors was measured every week until the third week when these mice were sacrificed, and the total weight of tumors was determined. Mouse experiments were performed following general guidelines issued by the Laboratory Animal Care Evaluation and Identification Association.

***Construction and sequencing of m^5^C-RIP libraries***

The m^5^C RNA immunoprecipitation sequencing (m^5^C-RIP-seq) service was provided by CloudSeq Inc. (Shanghai, China) ^3^. m^5^C-RIP-seq of mRNA, lncRNA and circRNA was performed in Huh7-sh-NC, Huh7-sh-ALYREF, HepG2-sh-NC and HepG2-sh-ALYREF cells. mRNA, lncRNA and circRNA were isolated from total RNA with oligo (dT) magnetic beads (ThermoFisher) according to the manufacturer’s instructions. RNA was randomly fragmented to approximately 200 nt. Protein A/G beads were incubated with m^5^C antibody by rotation at room temperature for 1 hour, coupling the beads and antibodies. Then, RNA fragments were incubated with bead-bound antibodies by rotation at 4°C for 4 hours, coupling the antibodies and RNA. The RNA/antibody complexes were then digested several times until the RNA was eluted from the complexes. RNA sequencing libraries were constructed using the GenSeq® Low Input Whole RNA Library Prep Kit (GenSeq, Inc.) according to the manufacturer’s protocol. A BioAnalyzer 2100 (Agilent Technologies, CA) was used to evaluate the libraries, and an Illumina NovaSeq platform was used for library sequencing.

***Sequencing data analysis***

The sequencing data analysis service was provided by CloudSeq Inc. (Shanghai, China). Raw data were produced using an Illumina NovaSeq 6000 sequencer, and the quality was controlled by Q30. Next, cutadapt software (v1.9.3) was used for trimming, removing low-quality reads and obtaining high-quality clean reads. STAR ^4^ software was used to align clean reads of input libraries with the reference genome (UCSC HG19), and mRNA, lncRNA and circRNA were identified by DCC ^5^ software. Clean reads were aligned to the reference genome using Hisat2 ^6^ software (v2.0.4). Then, methylated sites in each sample were identified using MACS ^7^ software. DiffReps ^8^ was used for the recognition of differentially methylated sites. Homemade scripts were used to screen peaks overlapping with the exons of mRNA, lncRNA and circRNA. GO and KEGG pathway enrichment analyses were performed to determine the genes of differentially methylated mRNAs, lncRNAs and circRNAs.

***RNA immunoprecipitation (RIP)***

The RIP assay was performed using an RNA immunoprecipitation Kit (Geneseed, Guangzhou, China) following the manufacturer’s instructions ^9^. A total of 1×10^7^ cells were lysed in complete lysis buffer, and RNA was extracted. The extracted RNA was incubated with streptavidin magnetic beads by rotation at 4°C for 3 hours. Then, ALYREF was added and incubated at 4°C for another 2 hours. IgG was used as a negative control. Finally, RNA was eluted and tested using mass spectrometry and Western blotting.

***Statistical analysis***

SPSS software (version 22.0, USA) and GraphPad Prism 8.0 (GraphPad Software, USA) were used to perform statistical analyses. Data are presented as the mean ± standard deviation (SD), and each experiment was independently repeated at least three times. The significance of differences between groups was evaluated using a two-tailed Student’s t test. The threshold for statistical significance was set at *P*< 0.05. **P* < 0.05; ***P* < 0.01; ****P* < 0.001.

**Supplementary Figures**


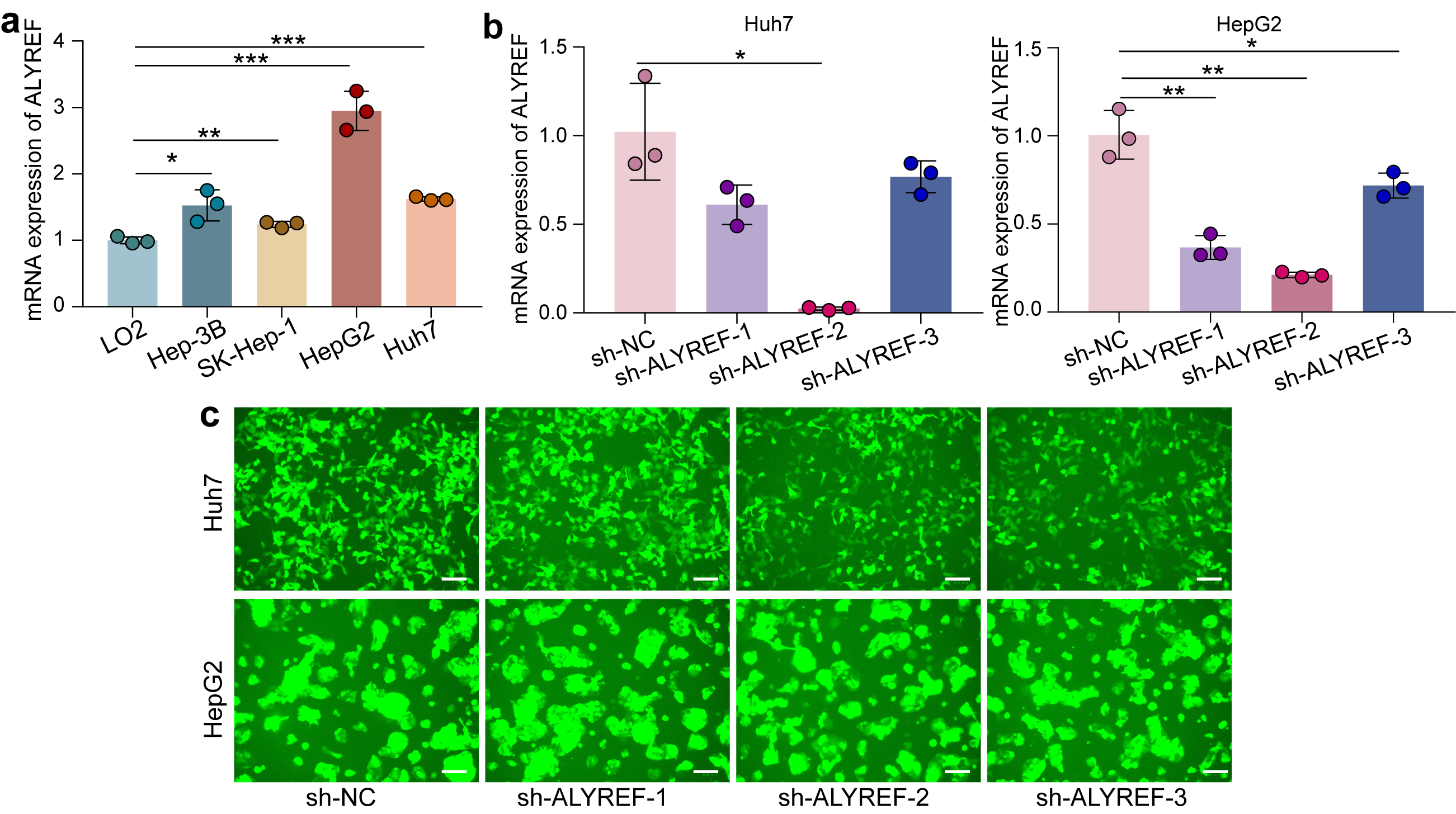


**Supplementary Fig. 1 The expression of ALYREF in various HCC cell lines and the testing knockdown efficiency of ALYREF. (a)** ALYREF expression in Hep-3B, SK-Hep-1, HepG2 and Huh7 cell lines was higher than that in the normal liver cell line LO2. **(b-c)** qRT-PCR and cell-based fluorescence assays showed the efficiency of ALYREF knockdown in Huh7 and HepG2 cells, and we adopted sh-ALYREF-2 for further study. Scale bar, 100 μm. **P* < 0.05; ***P* < 0.01; ****P* < 0.001.


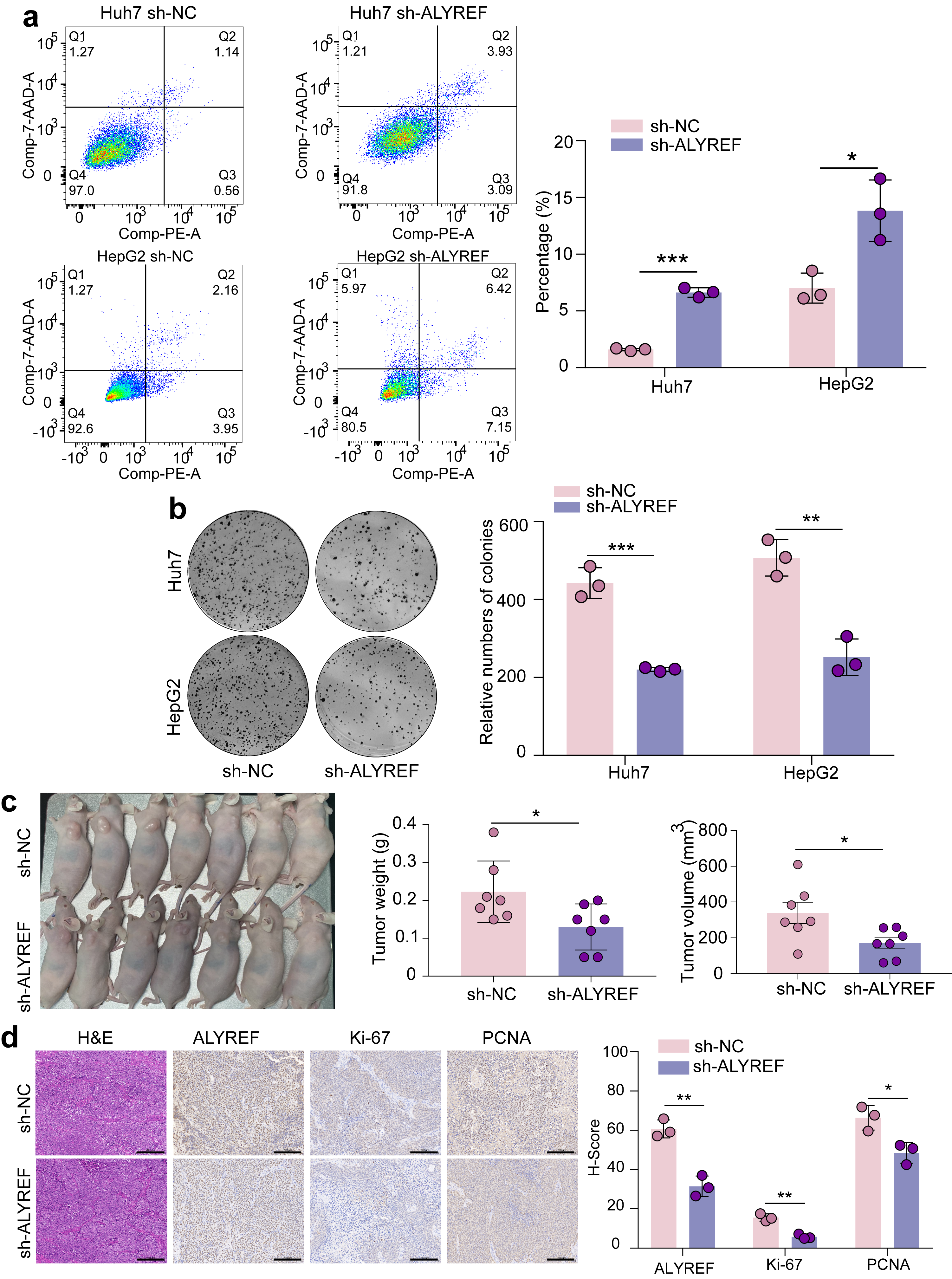


**Supplementary Fig. 2 sh-ALYREF inhibits HCC cell growth and increases tumor cell apoptosis in vitro and vivo. (a-b)** Cell apoptosis and proliferation were examined by flow cytometry and colony formation assay. **(c)** In vivo experiments on nude Balb/c mice displayed a smaller size and lighter weight of sh-ALYREF tumors compared to sh-NC tumors. **(d)** The liver tissues sections from BALB/c nude mice were stained with H&E and antibody that is specific for ALYREF, Ki67, and PCNA, respectively. Scale bar, 200 μm. **P* < 0.05; ***P* < 0.01; ****P* < 0.001.


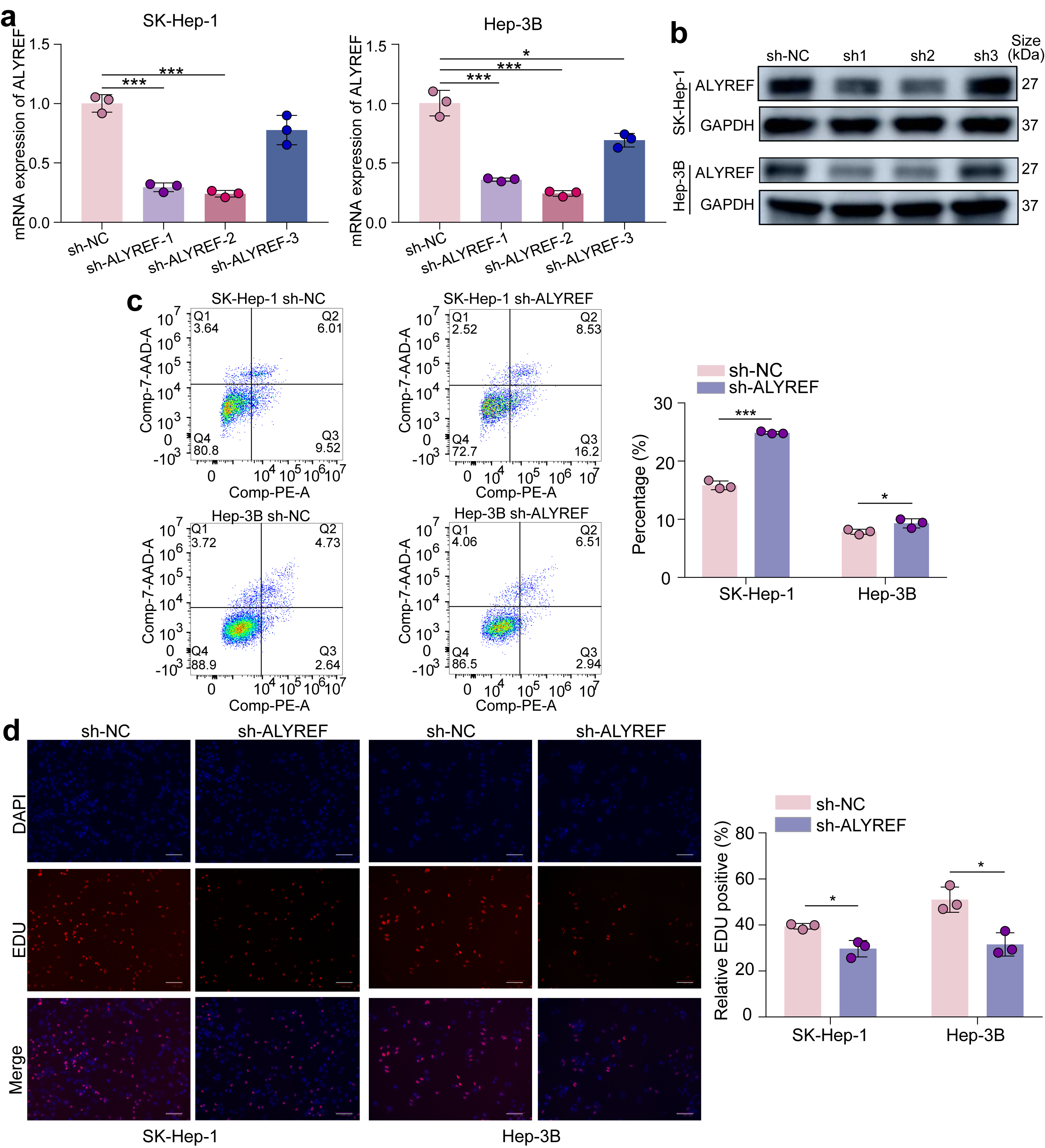


**Supplementary Fig. 3 sh-ALYREF inhibits HCC cell proliferation and increases tumor cell apoptosis in SK-Hep-1 and Hep-3B cell lines.** **(a-b)** qRT-PCR and Western blotting showed the efficiency of ALYREF knockdown in SK-Hep-1 and Hep-3B cell lines, and we adopted sh-ALYREF-2 for further study. **(c)** Cell apoptosis were examined by flow cytometry. **(d)** EdU assay showed the impaired vitality of ALYREF-deficient SK-Hep-1 and ALYREF-deficient Hep-3B cell lines. Scale bar, 100 μm. **P* < 0.05; ***P* < 0.01; ****P* < 0.001.


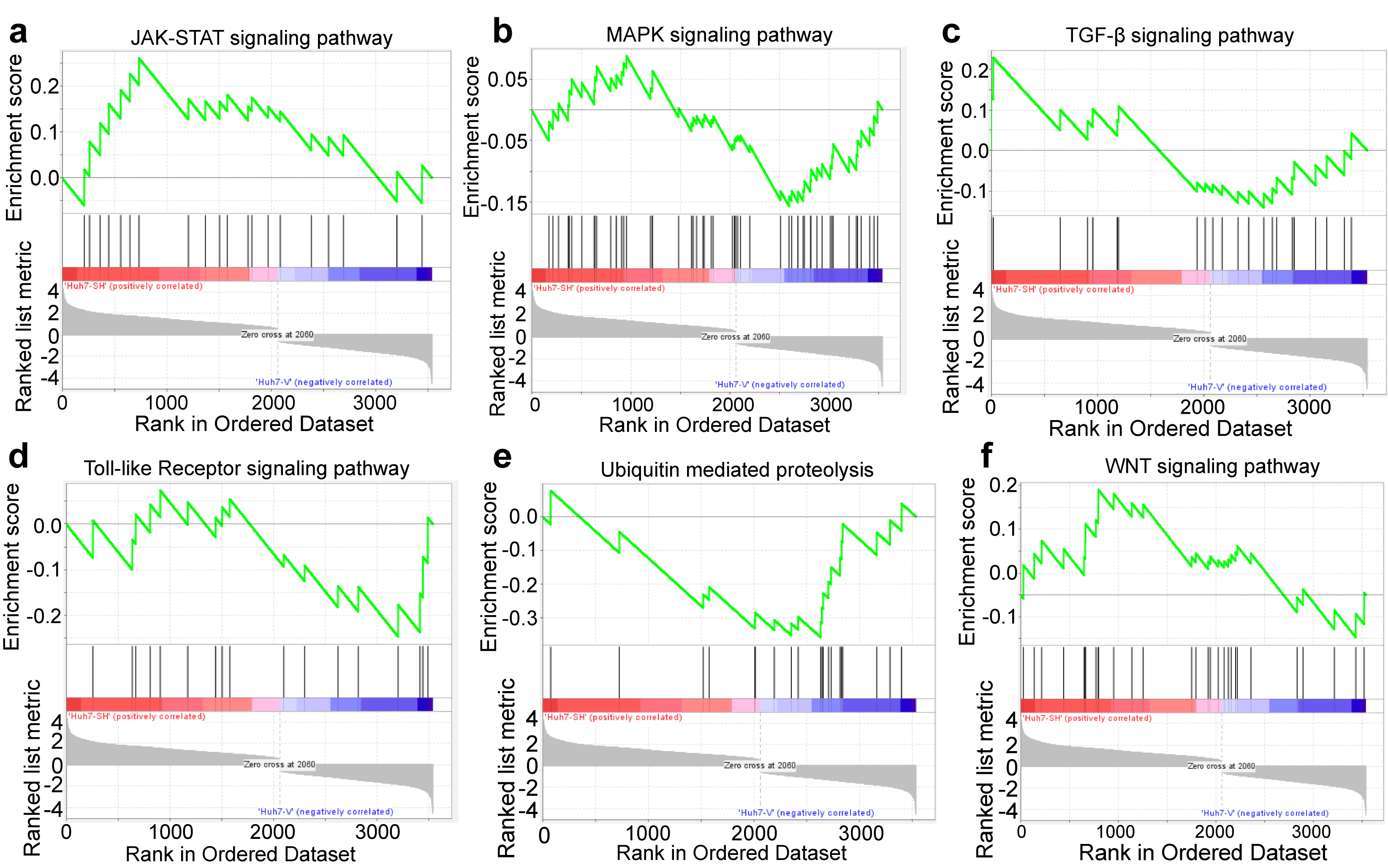


**Supplementary Fig. 4 GSEA of ALYREF-associated genes. (a-f)** GSEA performed on ALYREF-associated genes suggested the role of ALYREF in the JAK-STAT signaling pathway, MAPK signaling pathway, TGF-β signaling pathway, Toll-like receptor signaling pathway, ubiquitin-mediated proteolysis and WNT signaling pathway.


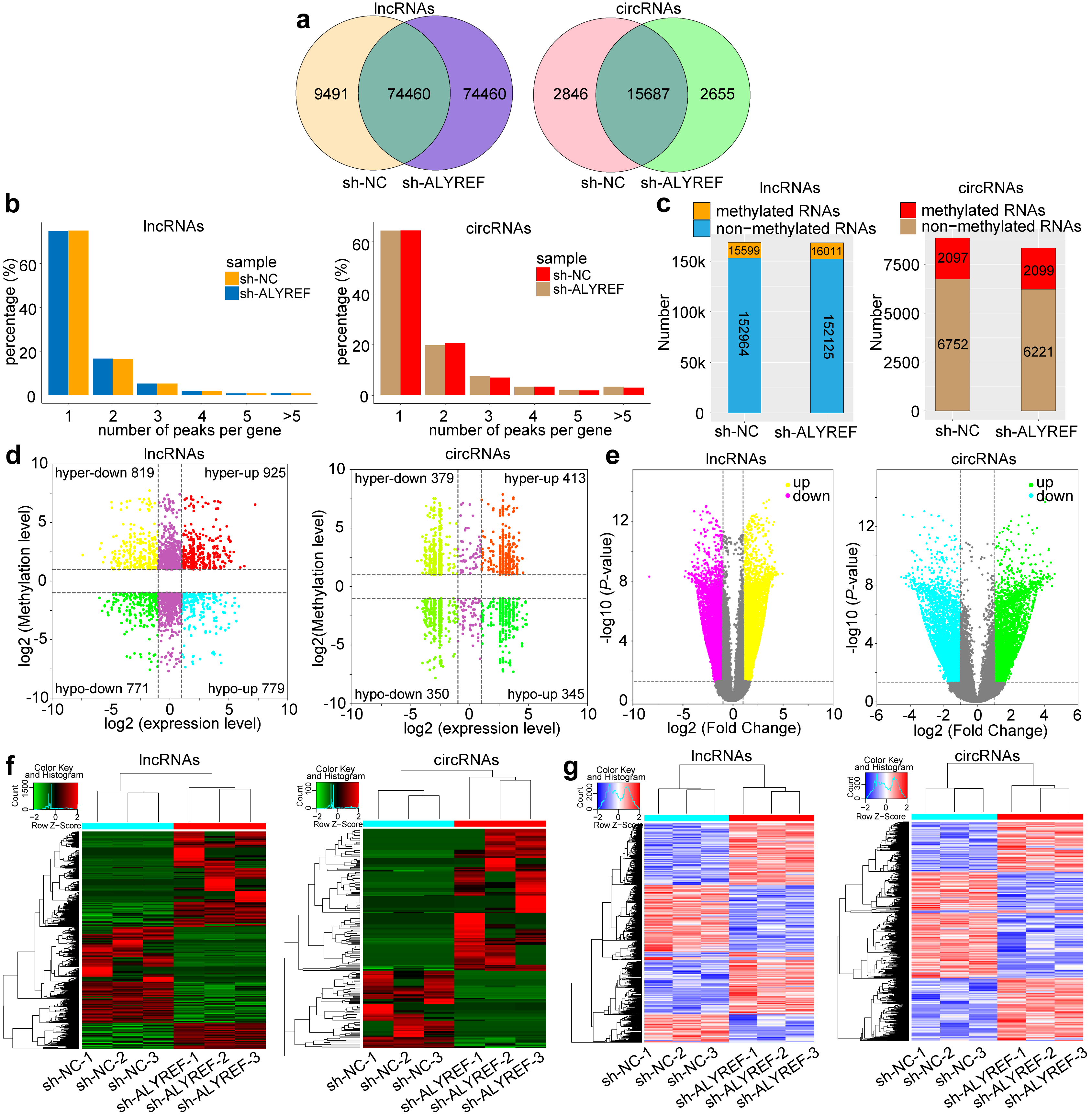


**Supplementary Fig. 5 m^5^C-MeRIP-Seq results of circRNAs and lncRNAs in both Huh7-sh-NC and Huh7-sh-ALYREF cells. (a)** m^5^C-MeRIP-Seq illustrated a total of 83,951 and 148,920 m^5^C sites of lncRNAs in Huh7-sh-NC and Huh7-sh-ALYREF cells, respectively. Additionally, 18,533 and 18,342 m^5^C sites of circRNA were present in Huh7-sh-NC and Huh7-sh-ALYREF cells, respectively. **(b)** Most lncRNAs and circRNAs possessed only one methylation peak in both Huh7-sh-NC and Huh7-sh-ALYREF cells. **(c)** The proportion of nonmethylated genes and methylated genes in lncRNAs and circRNAs. **(d)** Two four-quadrant diagrams displaying alterations in the methylation and expression of lncRNAs and circRNAs in Huh7-sh-ALYREF cells compared to Huh7-sh-NC cells. **(e)** Two volcano charts displaying changes in the expression levels of lncRNAs and circRNAs in Huh7-sh-ALYREF cells compared to Huh7-sh-NC cells. **(f)** Two heatmaps displaying the results of transcriptome hierarchical cluster analysis of lncRNAs and circRNAs in Huh7-sh-NC and Huh7-sh-ALYREF cells. **(g)** Two heatmaps show the results of hierarchical cluster analysis of both differentially methylated and differentially expressed lncRNAs and circRNAs in Huh7-sh-NC and Huh7-sh-ALYREF cells.


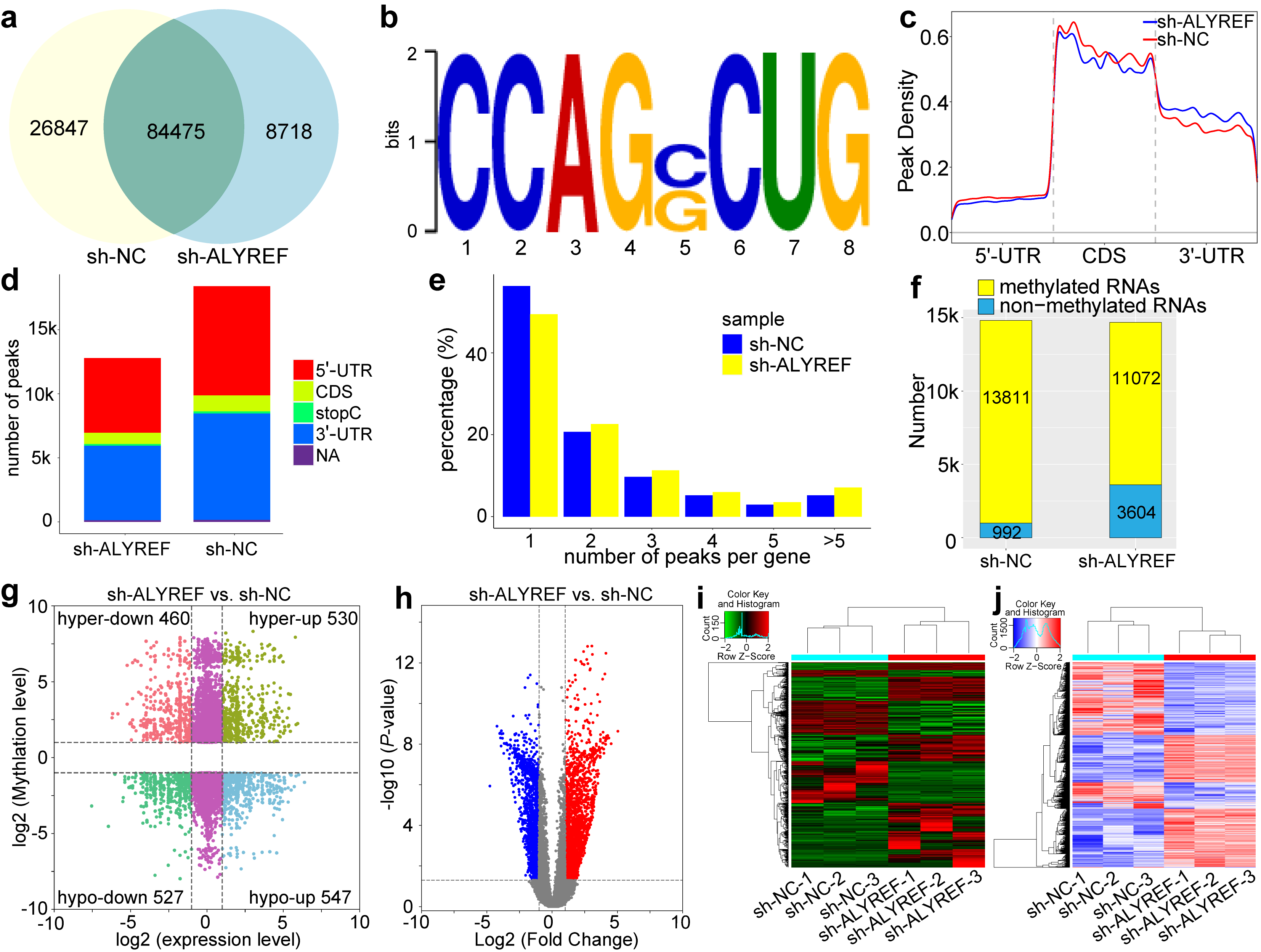


**Supplementary Fig. 6** **m^5^C-MeRIP-Seq results of mRNA in both HepG2-sh-NC and HepG2-sh-ALYREF cells. (a)** m^5^C-MeRIP-Seq illustrated a total of 111,322 and 93,193 m^5^C sites of mRNA in HepG2-sh-NC and HepG2-sh-ALYREF cells, respectively. Additionally, the two groups shared a majority of methylated mRNA sites. **(b)** The most conserved motif was CCAGRCUG (R=C/G) in both HepG2-sh-NC and HepG2-sh-ALYREF cells, which is the same as that in Huh7 cells. **(c-d)** In both HepG2-sh-NC and HepG2-sh-ALYREF cells, the CDS region had the highest peak density, followed by the 3’-UTR and 5’-UTR, but the number of peaks in the CDS region was smaller than that in the two other regions. **(e)** Most mRNAs possessed only one methylation peak in both HepG2-sh-NC and HepG2-sh-ALYREF cells. **(f)** A total of 13,811 and 11,072 methylated mRNAs and 992 and 3,604 nonmethylated mRNAs were found in HepG2-sh-NC and HepG2-sh-ALYREF cells, respectively. **(g)** A four-quadrant diagram displaying alterations in mRNA methylation and expression, showing 460 hypermethylated downregulated mRNAs, 530 hypermethylated upregulated mRNAs, 527 hypomethylated downregulated mRNAs and 547 hypomethylated upregulated mRNAs in HepG2-sh-ALYREF cells. **(h)** A volcano chart displaying changes in mRNA expression levels in HepG2-sh-ALYREF cells compared to HepG2-sh-NC cells. **(i)** A heatmap displaying the results of transcriptome hierarchical cluster analysis in HepG2-sh-NC and HepG2-sh-ALYREF cells. **(j)** A heatmap showing the results of hierarchical cluster analysis of differentially methylated mRNAs in HepG2-sh-NC and HepG2-sh-ALYREF cells.


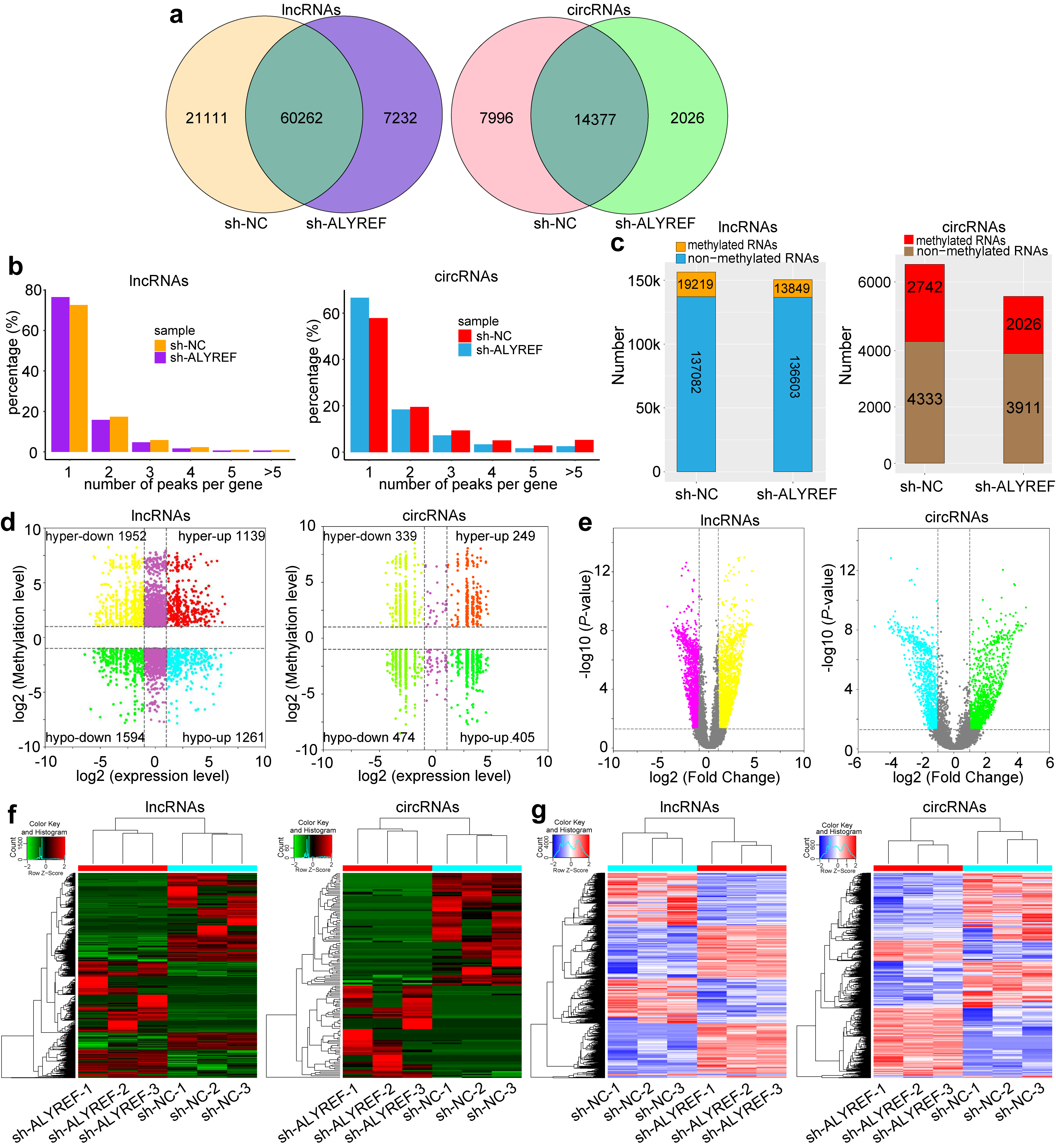


**Supplementary Fig. 7** **m^5^C-MeRIP-Seq results of circRNAs and lncRNAs in both HepG2-sh-NC and HepG2-sh-ALYREF cells. (a)** m^5^C-MeRIP-Seq illustrated a total of 81,373 and 67,494 m^5^C sites of lncRNA in HepG2-sh-NC and HepG2-sh-ALYREF cells, respectively. Additionally, 22,373 and 16,403 m^5^C sites of circRNA were present in HepG2-sh-NC and HepG2-sh-ALYREF cells, respectively. **(b)** Most lncRNAs and circRNAs possessed only one methylation peak in both HepG2-sh-NC and HepG2-sh-ALYREF cells. **(c)** Methylated genes and nonmethylated lncRNAs and circRNAs were tested in HepG2-sh-NC and HepG2-sh-ALYREF cells, respectively. **(d)** Two four-quadrant diagrams displaying alterations in the methylation and expression of lncRNAs and circRNAs in Huh7-sh-ALYREF cells compared to Huh7-sh-NC cells. **(e)** Two volcano charts displaying changes in the expression levels of lncRNAs and circRNAs in HepG2-sh-ALYREF cells compared to HepG2-sh-NC cells. **(f)** Two heatmaps displaying the results of transcriptome hierarchical cluster analysis of lncRNAs and circRNAs in HepG2-sh-NC and HepG2-sh-ALYREF cells. **(g)** Two heatmaps show the results of hierarchical cluster analysis of both differentially methylated and differentially expressed lncRNAs and circRNAs in HepG2-sh-NC and HepG2-sh-ALYREF cells.


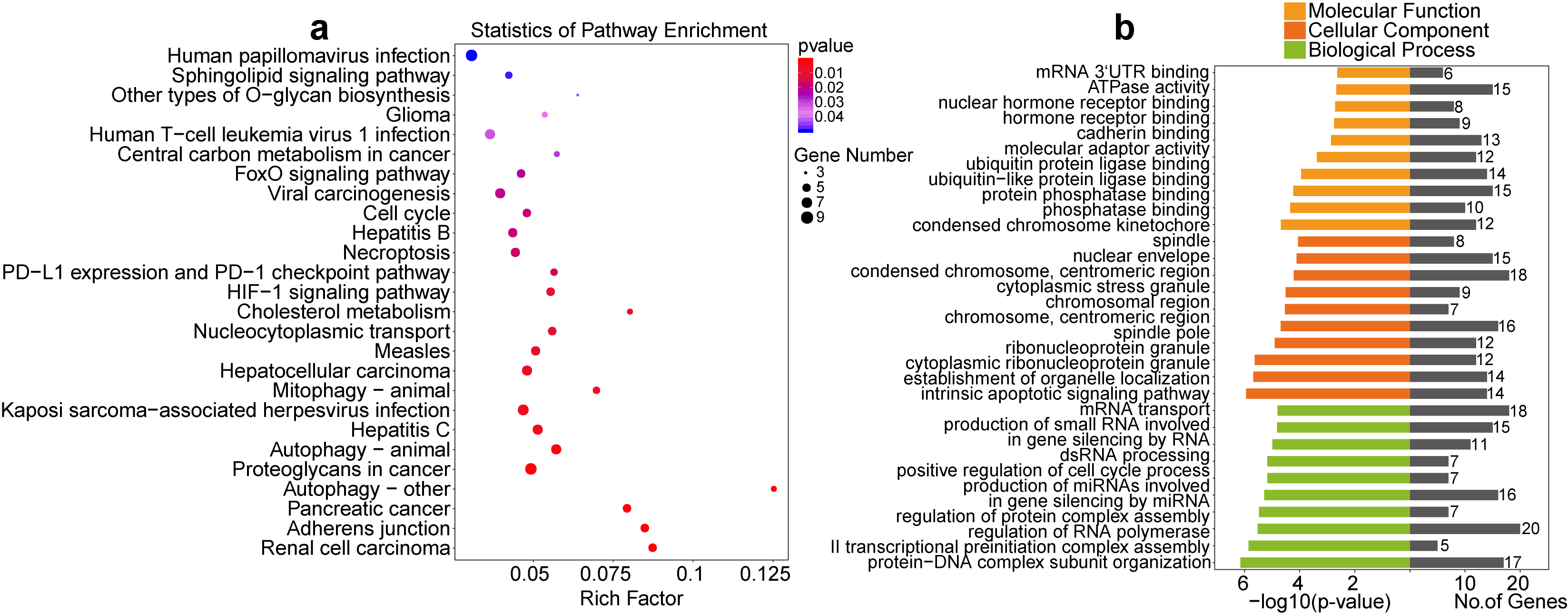


**Supplementary Fig. 8 The enrichment analysis of ALYREF-binding genes obtained from RIP. (a)** Results of KEGG enrichment analysis for ALYREF-binding genes. **(b)** Results of GO enrichment analysis for ALYREF-binding genes.

**References**

1. Xue C. et al. Intratumoral bacteria interact with metabolites and genetic alterations in hepatocellular carcinoma. *Signal Transduct Target Ther*. **7**, 335 (2022).

2. Liu H., Wang J., Zhang Y., Fan Y. & Wang K. Prognostic potential of the small GTPase Ran and its methylation in hepatocellular carcinoma. *Hepatobiliary Pancreat Dis Int*. **21**, 248-256 (2022).

3. Cui, X. *et al.* 5-Methylcytosine RNA Methylation in Arabidopsis Thaliana. *Mol Plant*. **10**, 1387-1399 (2017).

4. Dobin, A. *et al.* STAR: ultrafast universal RNA-seq aligner. *Bioinformatics*. **29**, 15-21 (2013).

5. Cheng, J., Metge, F. & Dieterich, C. Specific identification and quantification of circular RNAs from sequencing data. *Bioinformatics*. **32**, 1094-1096 (2016).

6. Kim, D., Langmead, B. & Salzberg, S. L. HISAT: a fast spliced aligner with low memory requirements. *Nat Methods*. **12**, 357-360 (2015).

7. Zhang, Y. *et al.* Model-based analysis of ChIP-Seq (MACS). *Genome Biol*. **9**, R137 (2008).

8. Shen, L. *et al.* diffReps: detecting differential chromatin modification sites from ChIP-seq data with biological replicates. *PLoS One*. **8**, e65598 (2013).

9. Keene J. & Komisarow J, Friedersdorf MB. RIP-Chip: the isolation and identification of mRNAs, microRNAs and protein components of ribonucleoprotein complexes from cell extracts. *Nat Protoc*. **1**, 302-307 (2006).
